# Prevalence and factors associated with restless legs syndrome among pregnant women in middle-income countries: a systematic review and meta-analysis

**DOI:** 10.3389/fmed.2023.1326337

**Published:** 2023-12-21

**Authors:** Esuyawkal Mislu, Betel Assalfew, Mulugeta Wodaje Arage, Fiker Chane, Tilahun Hailu, Lebeza Alemu Tenaw, Atitegeb Abera Kidie, Henok Kumsa

**Affiliations:** ^1^School of Midwifery, College of Health Science, Woldia University, Woldia, Ethiopia; ^2^School of Public Health, College of Health Science, Woldia University, Woldia, Ethiopia

**Keywords:** restless legs syndrome, systematic review, meta-analysis, sleep quality, RLS

## Abstract

**Introduction:**

Restless legs syndrome (RLS) is a debilitating condition characterized by uncomfortable sensations in the legs, typically occurring during periods of rest or sleep. It is more prevalent during pregnancy and is linked to sleep disturbances, diminished quality of life, and pregnancy complications. However, previous studies yielded inconsistent findings among pregnant women in middle-income countries. Consequently, this systematic review and meta-analysis sought to determine the pooled prevalence of restless legs syndrome and its associated factors in these populations.

**Method:**

A systematic review and meta-analysis was conducted on published studies from middle-income countries until May 2023. The review strictly adhered to the Preferred Reporting Items for Systematic Reviews and Meta-Analyses (PRISMA) guidelines. Relevant search terms were used to search for studies in PubMed, MEDLINE, EMBASE, and Google Scholar. Data extraction was performed using the Joanna Briggs Institute tool for prevalence studies. The meta-analysis was conducted using STATA 17 software, and heterogeneity was assessed using the *I*^2^ test, while publication bias was evaluated using Egger's test. Forest plots were also used to present the pooled prevalence and odds ratio (OR) with a 95% confidence interval (CI) using the random-effects model.

**Result:**

This review included 22 studies from nine countries with a total of 17, 580 study participants. The overall pooled prevalence of RLS among pregnant women in middle-income countries was 13.82% (95% CI: 13.31, 14.32), and having low hemoglobin level (AOR: 1.68, 95% CI: 1.29, 2.18), history of RLS (AOR: 7.54, 95% CI: 3.02, 18.79), muscle cramps (AOR: 3.58, 95% CI: 1.21, 10.61), excessive day time sleepiness (AOR: 4.02, 95% CI: 1.34, 12.04), preeclampsia (AOR: 2.06, 95% CI: 1.28, 3.30), and taking prophylactic iron supplementation (AOR: 0.59, 95% CI: 0.50, 0.69) were the identified factors associated with it.

**Conclusion:**

Generally, nearly one in every eight pregnant women in middle-income countries develop restless legs syndrome during pregnancy. Having low hemoglobin level, a history of RLS, muscle cramps, excessive daytime sleepiness, preeclampsia, and taking prophylactic iron supplementation were the identified factors associated with it. These findings underscore the importance of addressing the identified factors associated with RLS in order to effectively mitigate its occurrence among pregnant women.

## Introduction

Restless legs syndrome (RLS) is a syndrome characterized by uncomfortable sensations in the legs, particularly during sleep or rest, leading to an urge to move the legs. Although patients often have difficulty articulating their symptoms comprehensively, restless legs syndrome (RLS) typically manifests in the thighs, legs, and feet. It can be accompanied by sensations such as tingling, chilling, itching, crushing, and burning in the lower extremities (1, 2). This debilitating condition has affected individuals throughout the history (3). The diagnosis of RLS is difficult as it relies on clinical history, and there are other conditions, such as nocturnal leg cramps and hypnic jerks, that can be mistakenly diagnosed as RLS. However, a comprehensive history and physical examination can help differentiate the condition (3–5).

It is important to recognize that RLS is not solely characterized by its symptoms and severity. RLS is associated with various pregnancy complications, including pregnancy-induced hypertension (6–8), cardiovascular disease (6–9), gestational diabetes (10, 11), sleep disorders (12, 13), poor quality of life, and depression (14–16). These issues can contribute to adverse pregnancy outcomes such as preterm birth (17, 18), miscarriage (19), low birth weight (8, 18), postpartum depression (20), and baby blues (9). Furthermore, RLS increases the risk of recurrence, chronicity, and cardiovascular disease (21, 22). Surprisingly, RLS also elevates the likelihood of anxiety disorders and learning disabilities in offspring (14, 20, 23–25).

The prevalence of RLS has been estimated to range from 3.9 to 15% (22, 26, 27), with 2.5% of adults experiencing symptoms severe enough to require medical intervention (26). Women are two to three times more likely to have RLS compared to men (27–30), and the prevalence may increase to 25% during pregnancy (31).

Factors associated with RLS include female gender, pregnancy, lower socioeconomic status, poor health, low iron levels, and advanced age, comorbidity with Parkinson's disease or psychiatric disorders, and family history of similar disorders (16, 32). During pregnancy, variables such as taking prophylactic iron supplementation (11, 33), a history of premenstrual syndrome (9), chronic diabetes mellitus (21), later gestational age (34), poor sleep quality, stressful life events, and excessive daytime sleepiness (7) have also been associated with RLS.

However, previous studies have yielded inconsistent findings among pregnant women in middle-income countries. Furthermore, restless legs syndrome (RLS) is influenced by various factors such as lifestyle, previous experiences, and the quality of healthcare services received, which can vary significantly among individuals in middle-income countries compared to others. Therefore, the purpose of this systematic review and meta-analysis was to determine the pooled prevalence of RLS and identify the associated factors among pregnant women in middle-income countries.

## Methods

### Study design and search strategy

This systematic review and meta-analysis utilized published studies to determine the prevalence of RLS and its associated factors among pregnant women in middle-income countries. Various databases including PubMed, EMBASE, Google Scholar, CINAHL, Medline, SCOPUS, and reference lists were searched for relevant articles. The Preferred Reporting Items for Systematic Reviews and Meta-Analyses (PRISMA) guidelines and the Joanna Briggs Institute (JBI) Critical Appraisal Checklist were strictly followed in the review process to assess its quality.

The search for articles was performed with the following MeSH terms “((((((((((((prevalence) OR (magnitude)) OR (level)) AND (predictors)) OR (associated factors)) OR (determinant factors)) AND (restless leg syndrome)) OR (RLS)) OR (Willis-Ekbom disease)) OR (WED)) AND (antepartum)) OR (pregnancy)) OR (prenatal period)”. Boolean operators (AND/OR) were used to combine the different search terms and develop a search syntax. To boost the possibility of finding pertinent empirical research, additional sources (i.e., reference lists of included studies and systematic reviews of published articles) were manually searched. Moreover, the searches were made by combining the abovementioned MeSH term with the names of all countries in middle-income countries.

### Study selection and eligibility criteria

**Figure 1:**
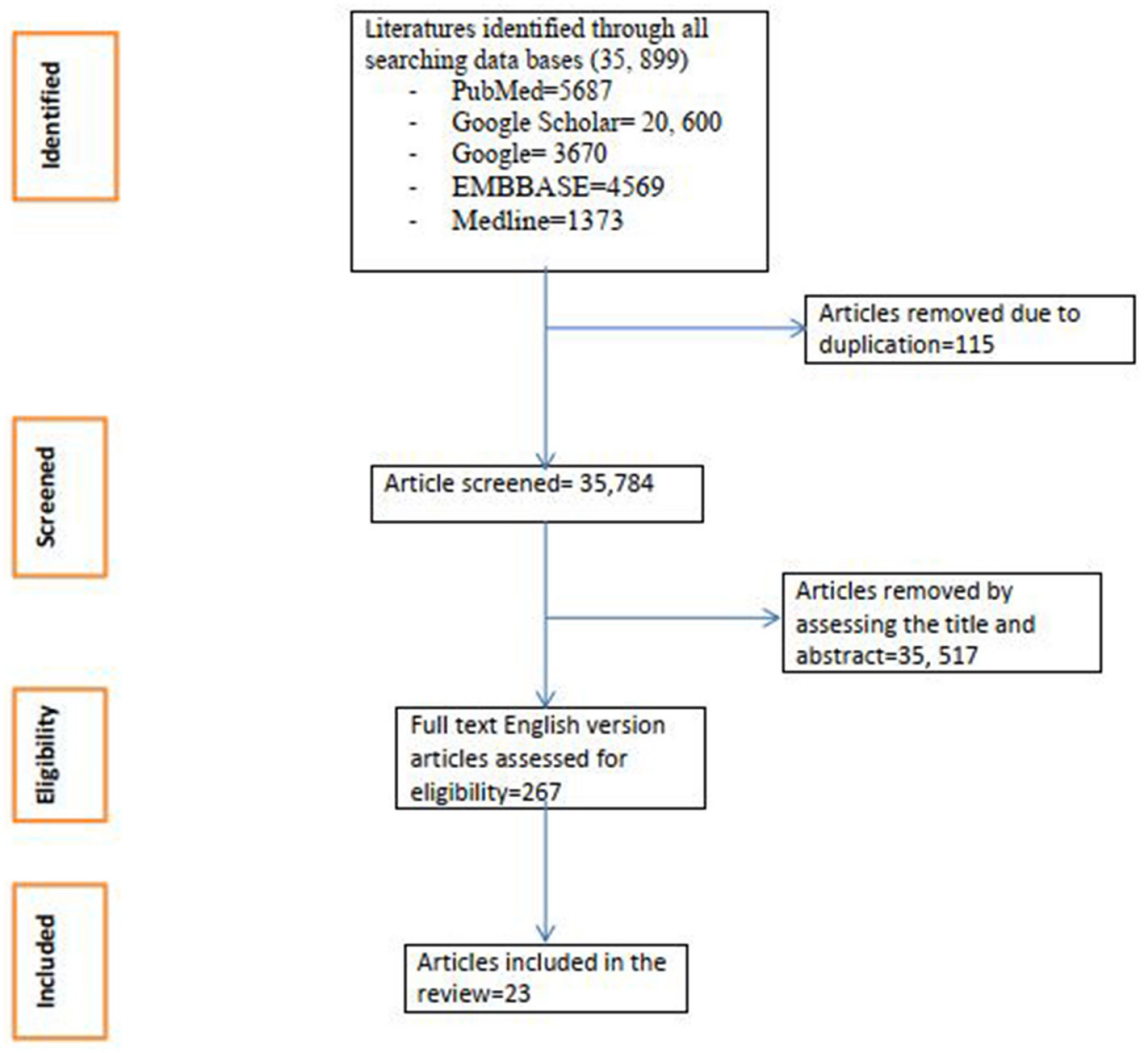
Flow chart of study selection for systematic review and meta-analysis of prevalence and associated factor for restless legs syndrome in middle income countries.

Peer-reviewed published articles written in English before April 2023 were included. This review included cross-sectional and case–control studies on RLS among pregnant women. However, case reports, case series, editorials, and studies published as abstracts were excluded. The references of selected articles were also screened to retrieve any additional articles. There were no restrictions on participant characteristics (Figure 1).

### Study outcomes

Prevalence and factors associated with RLS.

### Quality assessment

Articles were screened using their titles, abstracts, and full paper reviews prior to inclusion for meta-analysis. All authors assessed the quality of the included articles with the Joanna Briggs Institute (JBI) Critical Appraisal Checklist, which consists of eight total questions. Studies scoring five or more out of eight on the JBI criteria were considered of good quality and included in the review. Discrepancies in the critical appraisal process among authors were resolved through discussion. JBI result of the included studies is available in the Supplementary material.

### Data extraction and management

The data extraction tool contained information regarding the author, year of publication, study area and region, study design, study population, sample size, response rate, outcome measured, prevalence, and factors associated with RLS among pregnant women. Furthermore, each author was independently examined, and an agreement was reached among all authors on the titles and abstracts to be incorporated into this review and meta-analysis.

### Registration and protocol

This review did not undergo prior registration or protocol preparation. Therefore, no modifications or adjustments were made to the review process.

### Heterogeneity and publication bias

The heterogeneity among included studies was assessed by using the *I*^2^ statistics, with a *p*-value < 0.05 indicating the presence of heterogeneity. Based on *I*^2^ test statistics findings, heterogeneity among the included studies was categorized as low (25%), moderate (50%), and high (75%). Moreover, publication bias was also assessed using the Egger regression asymmetry test, with a p-value < 0.05 suggesting the presence of publication bias. The Duval and Tweedie non-parametric trim-and-fill analysis was conducted to observe the presence of publication bias using the random-effects analysis.

### Data processing and analysis

Data were entered into Microsoft Excel, and the meta-analysis was conducted using STATA 16 software. Forest plots were used to present the results of the meta-analysis. The random-effects model of analysis was used as a method of meta-analysis. Moreover, the meta-analysis regression was conducted to identify the sources of heterogeneity among studies. It was conducted in a classified study setting and region wise in the included studies. Predictors of restless legs syndrome were presented using odds ratios at a 95% confidence interval (CI).

## Result

### Characteristics of included studies

A total of 23 studies were included in this review. A single study was included from Nigeria (35), Thailand (36), Peru (37), and Brazil (38) each. Two studies were from India (39, 40), three were from Pakistan (41–43), four from Iran (17, 44–46), four from China (7, 8, 47, 48), and five from Turkey (49–53). According to WHO region classification studies included for this review, one was from the African region (35), three from the south-east region (36, 39, 40), five from the European region (49–53), four from the western pacific region (7, 8, 47, 48), two from the American region (37, 38), and eight from the Eastern Mediterranean (EM) region (17, 41–46). The World Bank's classification for lower- and upper-middle-income countries, which was based on the gross national income per capita of the countries (from $1,136 to 4,465, and $4,466 to 13,845, respectively), was utilized (54, 55). Twelve studies (7, 8, 36–38, 47–53) were from upper-middle-income countries, and ten studies were (17, 35, 39–46) from lower-middle-income countries. Except for a case–control study from Iran (56), all others were cross-sectional.

### Prevalence of restless legs syndrome among pregnant women

The pooled prevalence of RLS in this review is 13.82 (95% CI: 13.31, 14.32). The *I*^2^ test was 96.2% with a *p*-value < 0.0001, which indicated significant heterogeneity. The forest plot showed the overall and individual effect size of the studies. Publication biases among the included studies were examined by using funnel plots and Egger's regression test. The results of funnel plots showed an asymmetric shape, which indicates the presence of publication bias among included studies. Supplementary Figure 1 contains the funnel plot.

### Subgroup analysis

**Figure 2:**
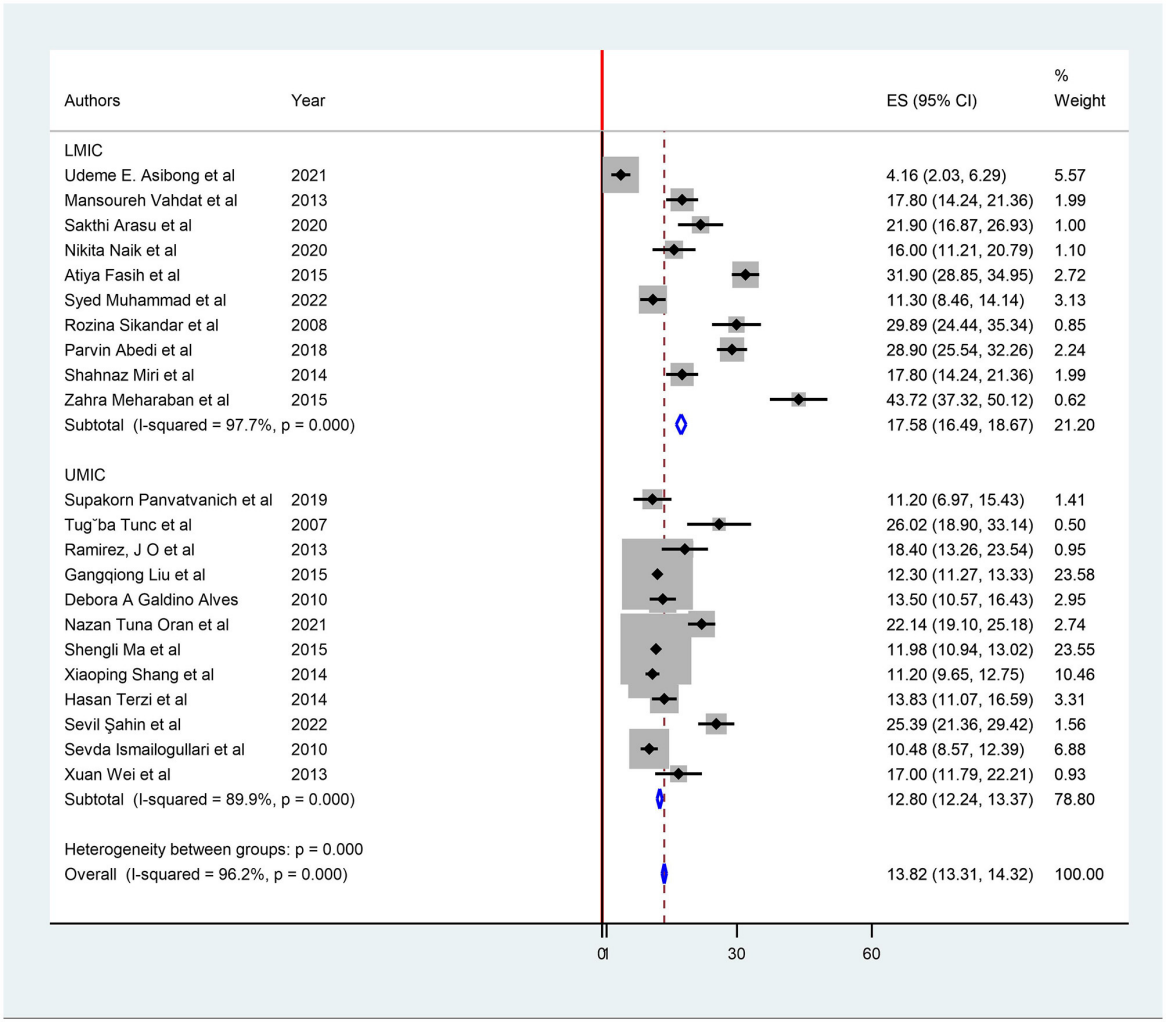
Subgroup analysis based on lower vs. upper middle-income countries for restless legs syndrome prevalence.

The subgroup analysis was performed based on WHO regions and the World Bank's classification of countries. Table 1 shows the pooled prevalence, and it was found to be the highest in the Eastern Mediterranean region at 22.91% (95% CI: 21.54, 24.27), and lowest in Africa, 4.16 % (95% CI: 2.03, 6.29), based on the WHO region classification. Moreover, subgroup analysis showed that the highest pooled prevalence of RLS was seen in lower-middle-income countries, 17.58 (95% CI: 16.49, 18.67), compared to upper-middle-income countries, 12.80% (95% CI: 12.24, 13.37) (Table 1) (Figure 2). Supplementary Figure 2 contains the forest plot for subgroup analysis based on the WHO region classification.

**Table 1:** Summary of subgroup analysis of the pooled prevalence of RLS among pregnant women in middle-income countries, 2023.

**Category type**	**Subgroups**	**Number of studies**	**Sample size**	***P* (%)**	**95% CI**	**Heterogeneity**	
						***I* ^2^**	***p*-value**
Country's category based on the WHO region	African Nigeria	1	338	4.16	2.03–6.29	–	–
	American Peru Brazil	2	742	14.70	12.15–17.24	62%	0.105
	EM Iran Pakistan	7	3,466	22.91	21.54–24.27	96.5%	0.000
	European Turkey	5	2,896	15.42	14.12–19.71	94.9%	0.000
	South-East Asia India Thailand	3	699	15.75	13.07–18.43	80.4%	0.006
	Western-pacific China	4	9,436	12.05	11.39–12.71	38.3%	0.182
Country's category based on income	LMIC Nigeria Pakistan India Iran	10	4,289	17.58	16.49–18.67	97.7%	0.000
	UMIC Brazil Peru Turkey Thailand China	12	13,291	12.80	12.24–13.37	89.9%	0.000

### Factors associated with restless legs syndrome among pregnant women

In this meta-analysis, pregnant women with low hemoglobin level were 1.68 times (AOR: 1.68, 95% CI: 1.29, 2.18) more likely to have RLS as compared to those with normal hemoglobin level. Similarly, women with history of RLS had 7.54 times (AOR: 7.54, 95% CI: 3.02, 18.79); women with muscle cramps had 3.58 times (AOR: 3.58, 95% CI: 1.21, 10.61); women with excessive daytime sleepiness had 4.02 times (AOR: 4.02, 95% CI: 1.34, 12.04); and women with preeclampsia had 2.06 (AOR: 2.06, 95% CI: 1.28, 3.30) times higher odds of developing RLS as compared to their counterparts. On the contrary, pregnant women who took prophylactic iron supplementation were 41% (AOR: 0.59, 95% CI: 0.50, 0.69) less likely to develop RLS as compared to those who did not take prophylactic iron supplementation (Table 2).

**Table 2:** Factors associated with RLS among pregnant women in middle-income countries, 2023.

**Variables**	**ES (OR)**	**95% CI (OR)**
Hgb level (low)	1.68	1.29, 2.18
History of RLS	7.54	3.02, 18.79
Muscle cramps	3.58	1.21, 10.61
Excessive daytime sleepiness	4.02	1.34, 12.04
Prophylactic iron supplementation	0.59	0.50, 0.69
Preeclampsia	2.06	1.28, 3.30
Low income	0.90	0.26, 3.19

## Discussion

The current meta-analysis revealed several factors that were highly and positively associated with RLS during pregnancy. These include having low hemoglobin levels, a history of RLS, muscle cramps, excessive daytime sleepiness, and preeclampsia. However, pregnant women who took prophylactic iron supplementation had a 41% reduced likelihood of developing RLS compared to those who did not take prophylactic iron supplementation.

The pooled prevalence of RLS during pregnancy was found to be 13.82%. This indicates a significantly higher prevalence of RLS among pregnant women compared to the global general population (3%) and among all women (4.7%) (57). The higher prevalence rate can be attributed to various factors such as hormonal differences, pressure on adjacent blood vessels and nerves by the fetus, psychological stressors, lifestyle changes during pregnancy, and physiological changes such as vascular changes and nutrient deficiencies (iron, calcium, and magnesium) (31, 58, 59).

However, the current pooled prevalence of RLS during pregnancy (13.82%) is lower than that reported in a meta-analysis conducted in Iran (32.9%) (60). This difference could be due to variations in the included studies. The Iran meta-analysis included studies conducted on both sexes and different health problems, including patients on hemodialysis (60). Additionally, the prevalence of RLS in middle-income countries is lower than the global prevalence among all trimesters (21%) (61) and the third trimester (22.9%) of pregnancy (34). This difference could be attributed to the difference in the study population as most of the studies included in the worldwide meta-analysis were from high-income countries. This could result in a higher likelihood of diagnosis among affluent women compared to those with comparatively limited access to healthcare, higher stress levels associated with certain lifestyles or occupations, and sedentary lifestyles. Additionally, advanced gestational age among third-trimester pregnant women might have contributed to higher RLS prevalence (45).

This systematic review and meta-analysis identified a significant association between RLS among pregnant women in middle-income countries and hemoglobin levels. Women with low hemoglobin levels were 1.68 times more likely to develop RLS as compared to those with normal hemoglobin levels. Similarly, pregnant women who took prophylactic iron supplementation were 41% less likely to develop RLS compared to those who did not. These findings are consistent with previous studies conducted on RLS and its association with iron and dopamine in mice, pregnant or non-pregnant women, and older adults (31, 58, 62–64).

Furthermore, studies have shown that RLS is related to dysfunction of the dopamine system, particularly in the brain regions that control body movement. Iron also plays a crucial role in the production of dopamine, a neurotransmitter involved in regulating movement and sensations in the body (62–64). Therefore, a lack of iron can impair the production and regulation of dopamine, contributing to the development or worsening of RLS (31, 58). Iron deficiency further exacerbates dysfunction leading to symptoms of RLS like the urge to move the legs, discomfort, and disturbed sleep (64–67). Increasing hemoglobin levels through prophylactic iron supplementation is advisable to restore normal dopamine levels and alleviate the symptoms of RLS in pregnant women (58, 64).

The current study also found that women with a history of RLS were 7.54 times more likely to have RLS in their current pregnancy. This could be due to hormonal fluctuations, especially estrogen and progesterone (31, 58, 68), iron deficiency, increased blood volume, and pressure of the gravid uterus on nerves. These factors might contribute to the recurrence of RLS (68).

Additionally, pregnant woman who experienced muscle cramps had 3.58 times higher odds of having RLS as compared to their counterparts. Although muscle cramps and RLS are two distinct conditions (69–71), they may share some similarities in terms of leg sensations (70, 71). Muscle cramps can occur because of muscle fatigue, dehydration, electrolyte imbalances, or overuse. The association between RLS and muscle cramps could be attributed to abnormal nerve activity, issues with circulation and blood flow, and electrolyte imbalances (low levels of potassium, magnesium, or calcium) (70).

RLS was found to be 4.02 times higher among pregnant women with excessive daytime sleepiness compared to those with normal sleeping patterns. This finding is supported by previous studies, which suggests that women with RLS may have poor sleep quality (72, 73), short duration of sleep at night (73–75), depression, and excessive daytime sleepiness for compensation (72, 76, 77).

Furthermore, pregnant woman with preeclampsia were 2.06 times more likely to develop RLS compared to their counterparts. This could be due to vascular changes and reduced blood flow associated with preeclampsia, which may contribute to the development or worsening of RLS symptoms (78–80). On the other hand, RLS, which is associated with obstructive sleep apnea, can result in the occurrence of preeclampsia by causing apneas or hypopneas, inducing sympathetic activation, endothelial dysfunction, and abnormal placental physiology (50, 81–85). This can be decreased by increasing ventilation and ensuring an open airway through lifestyle modifications, constant positive airway pressure, or other mechanisms based on individual needs (86, 87).

Additionally, a single study reported associations between RLS among pregnant women and various conditions, including poor sleep quality (40), higher Epworth sleep scale scores (7, 8, 36, 37), higher gestational age (45), caffeine consumption (45), smoking (8), hypertension (8), diabetes mellitus (8), gestational hypertension (45), increased wakefulness (48), increased number of children (52), menstrual irregularity before pregnancy (49), history of gynecologic surgery (49), thyroid disease (47), arthritis (47), varicose vein (47), and cesarean section (44).

It is important to acknowledge and consider certain limitations in these findings before generalizing the results. First, there was significant heterogeneity among the included studies, and this heterogeneity was not adequately addressed through subgroup analysis. Even though all studies were done among pregnant women, there was a difference in gestational age and obstetric complications. Additionally, it should be noted that only published articles were considered in this review, which might introduce publication bias. Future studies that explore the relationship between RLS and other factors such as hormonal levels, social functioning, and substance and alcohol use could provide valuable insights for a more comprehensive understanding of the topic.

## Conclusion

During pregnancy, approximately one out of every eight women in middle-income countries experience restless legs syndrome (RLS), a higher prevalence compared to the general population. This puts pregnant women at a greater risk for RLS. Various factors have been associated with RLS, such as low hemoglobin levels, a previous history of RLS, muscle cramps, excessive daytime sleepiness, and preeclampsia. However, taking prophylactic iron supplementation has been shown to reduce the likelihood of developing RLS. Therefore, it is crucial to take measures to prevent pre-eclampsia, address muscle cramps, provide prophylactic iron supplementation to prevent anemia, and promote sufficient sleep, thereby decreasing the risk of pregnant women developing RLS. Additionally, it is important to conduct further research and review follow-up studies to determine the prevalence and associated factors of RLS among women in low-income countries.

## Data availability statement

The original contributions presented in the study are included in the article/Supplementary material, further inquiries can be directed to the corresponding author.

## Author contributions

EM: Conceptualization, Data curation, Formal analysis, Investigation, Methodology, Resources, Software, Supervision, Validation, Visualization, Writing – original draft, Writing – review & editing. BA: Data curation, Investigation, Methodology, Resources, Writing – original draft. MA: Data curation, Investigation, Methodology, Resources, Visualization, Writing – original draft. FC: Data curation, Investigation, Methodology, Resources, Validation, Writing – original draft. TH: Data curation, Investigation, Methodology, Resources, Supervision, Writing – original draft, Writing – review & editing. LT: Data curation, Formal analysis, Investigation, Resources, Software, Supervision, Validation, Writing – original draft, Writing – review & editing. AK: Data curation, Formal analysis, Investigation, Methodology, Resources, Software, Supervision, Validation, Visualization, Writing – original draft, Writing – review & editing. HK: Data curation, Formal analysis, Investigation, Methodology, Resources, Software, Supervision, Validation, Visualization, Writing – original draft, Writing – review & editing.
