# Supplementary material for: Prevalence and factors associated with restless legs syndrome among pregnant women in middle-income countries: a systematic review and meta-analysis
Source: Front Med (Lausanne). 2023 Dec 21;10:1326337. doi: 10.3389/fmed.2023.1326337 (PMC10771314; doi:10.3389/fmed.2023.1326337)
Supplement: Supplementary file 1 [file Data_Sheet_1.docx]

Supplementary file


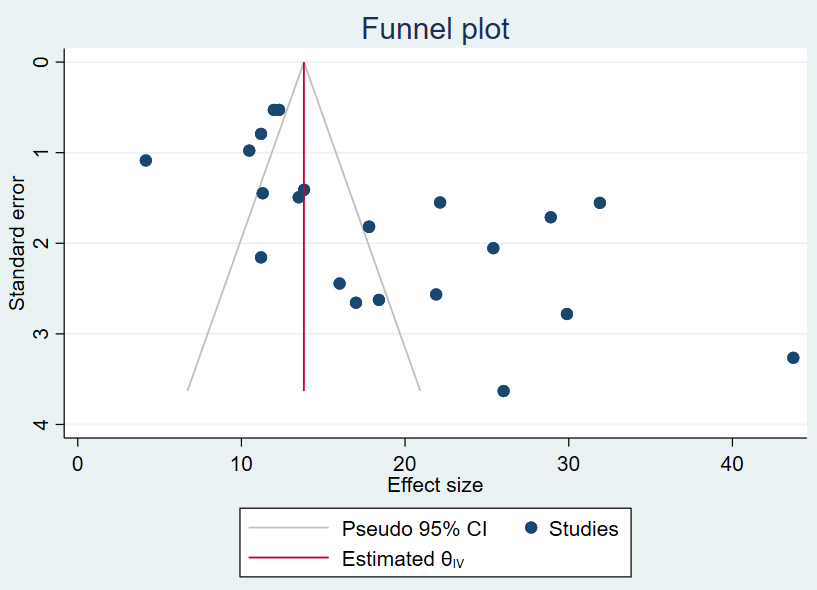


Figure 1: Funnel plot of publication bias test among the studies conducted on RLS in middle income counties

Figure 2: Forest plot of the pooled prevalence of RLS among pregnant women in middle income countries based WHO of countries classification
